# Gentamicin release from commercially-available gentamicin-loaded PMMA bone cements in a prosthesis-related interfacial gap model and their antibacterial efficacy

**DOI:** 10.1186/1471-2474-11-258

**Published:** 2010-11-10

**Authors:** Daniëlle Neut, Otto S Kluin, Jonathan Thompson, Henny C van der Mei, Henk J Busscher

**Affiliations:** 1 Department of Biomedical Engineering, University Medical Center Groningen and University of Groningen, Antonius Deusinglaan 1, 9713 AV Groningen, The Netherlands; 2 Department of Orthopedic Surgery, University Medical Center Groningen and University of Groningen, Hanzeplein 1, 9713 GZ Groningen, The Netherlands; 3 DePuy International Ltd, 1, White Rose Office Park, Millshaw Park Lane, Leeds LS11 0BG, UK

## Abstract

**Background:**

Around about 1970, a gentamicin-loaded poly (methylmethacrylate) (PMMA) bone cement brand (Refobacin Palacos R) was introduced to control infection in joint arthroplasties. In 2005, this brand was replaced by two gentamicin-loaded follow-up brands, Refobacin Bone Cement R and Palacos R + G. In addition, another gentamicin-loaded cement brand, SmartSet GHV, was introduced in Europe in 2003. In the present study, we investigated differences in gentamicin release and the antibacterial efficacy of the eluent between these four cement brands.

**Methods:**

200 μm-wide gaps were made in samples of each cement and filled with buffer in order to measure the gentamicin release. Release kinetics were related to bone cement powder particle characteristics and wettabilities of the cement surfaces. Gaps were also inoculated with bacteria isolated from infected prostheses for 24 h and their survival determined. Gentamicin release and bacterial survival were statistically analysed using the Student's t-test.

**Results:**

All three Palacos variants showed equal burst releases but each of the successor Palacos cements showed significantly higher sustained releases. SmartSet GHV showed a significantly higher burst release, while its sustained release was comparable with original Palacos. A gentamicin-sensitive bacterium did not survive in the high gentamicin concentrations in the interfacial gaps, while a gentamicin-resistant strain did, regardless of the type of cement used. Survival was independent of the level of burst release by the bone cement.

**Conclusions:**

Although marketed as the original gentamicin-loaded Palacos cement, orthopaedic surgeons should be aware that the successor cements do not appear to have the same release characteristics as the original one. Overall, high gentamicin concentrations were reached inside our prosthesis-related interfacial gap model. These concentrations may be expected to effectively decontaminate the prosthesis-related interfacial gap directly after implantation, provided that these bacteria are sensitive for gentamicin.

## Background

Deep infections in a total joint replacement are potentially catastrophic events for patients. Antibiotic-loaded PMMA bone cements (ALBCs) are used in joint replacement procedures to fix implants, with the antibiotic acting to reduce the risk of infection. Surgeons have been mixing antibiotics into bone cement, but mixing antibiotics intra-operatively into carefully composed bone cement formulas presents certain risks. For example, the surgeon can never be sure that the antibiotic is evenly distributed throughout the mixture, or that the mechanical properties of the cement will not be compromised. Commercially-available ALBC is guaranteed to be evenly blended, and has been shown to have higher release rates when compared to manually mixed cement [1].

Palacos bone cement with gentamicin added was the first used combination (formerly commercially available as Refobacin Palacos R) and surgeons have now used this bone cement for over 30 years. The production of Refobacin Palacos R (by: Heraeus GmbH; distributor: Biomet Merck/Biomet Europe) stopped in 2005 because of corporate reorganization. Subsequently, the two companies filled this blank with follow-up products: Refobacin Bone Cement R (distributed by Biomet Europe) and Palacos R + G (distributed by Heraeus GmbH). Essentially, both companies claim that the new cements are equivalent to their predecessor. In addition, SmartSet GHV (distributed by DePuy CMW) is a gentamicin-loaded bone cement introduced in Europe in 2003. There are studies published in which various properties (mechanical properties, handling characteristics, viscosity, volumetric shrinkage), and gentamicin elution of the three Palacos variants (Refobacin Palacos R, Refobacin Bone Cement R, and Palacos R + G) and SmartSet GHV were compared [2-5]. SmartSet GHV contains 1 g of active gentamicin, all three Palacos variants contain only 0.5 g of active gentamicin. Some studies, however, have shown that the amount of antibiotic incorporated does not necessarily determine the amount of release [6,7]. Moreover, the antibacterial efficacy of an ALBC is not entirely determined by the kinetics of release of the antibiotic [8].

Numerous *in vitro *studies on antibiotic release from ALBCs have been published [1,6,7], but a major drawback of these studies is that they do not account for a clinically realistic volume-to-area ratio; that is, the antibiotic concentrations reached are much lower than can be achieved clinically due to release into a too large fluid volume in relation to the cement area from which antibiotic is released. Thus, the purpose of the present *in vitro *study was to investigate whether there are any differences between the four gentamicin-loaded cements in terms of their antibiotic release and antibacterial efficacy in a prosthesis-related interfacial gap model [9,10], that simulates the interfacial gap that occurs *in vivo *between bone cement and the bone or prosthesis [11]. Cement powder properties and the wettability of the cured cement were determined and related to the release kinetics of the gentamicin.

Furthermore, the influence of the release of the gentamicin released on survival of bacteria in specimens of the cured cements was determined.

## Methods

### Bone cements

Four commercially-available gentamicin-loaded PMMA bone cement brands were used in this study: Refobacin Palacos R (Biomet Merck/Biomet Europe, Germany), Refobacin Bone Cement R (Biomet Europe, Germany), Palacos R + G (Heraeus Medical GmbH, Germany), and SmartSet GHV (DePuy CMW, England).

### Characterization of cement powder

The morphology, size and shape of the particles of the cement powders were determined using a field emission scanning electron microscope (Type 6301F; JEOL, Japan). The powder was sputter-coated with gold/palladium (~3 nm) and examination was done at an acceleration voltage of 2.0 kV.

The particle size distribution of the powder was determined using a laser diffraction apparatus (Sympatec HELOS; Sympatec Gm Clausthal-Zellerfeld, Germany), using a RODOS dry powder disperser (at 3.0 bar). A lens of 200 mm was used and calculations were based on the Fraunhofer diffraction theory. All data given are the mean of three measurements.

### Wettability of bone cements

To establish the wettability of the four bone cement surfaces, advancing type contact angles were measured by putting 1 μl water droplets on a cured cement specimen. Water droplets were observed with a video-camera, connected to a contour-monitor for observer independent readings. Four specimens were analyzed per cement, putting two droplets on each specimen.

### Preparation of the interfacial gap model

The preparation of the bone cements started with mixing the powder with the liquid, according to the manufacturer's instructions. This was performed manually with a spatula in a ceramic bowl, under atmospheric pressure and at ambient temperature. At the time specified for the start of application, as stated in the respective manuals, the cement was spread over a polytetrafluoroethylene (PTFE) mould. Prior to this, the mould was fitted with stainless-steel strips with a thickness of 200 μm, as described before [9,10]. The thickness of the strip was chosen on the basis of work by Wang et al. [11], showing that the boundary layer between bone cement and bone was 50 to 500 μm wide along 15% of the interfacial circumference in the femur of a cadaver pig. This prosthesis-related gap matters most in implant infection, because it is considered to be an immuno-incompetent zone [12].

After application of the cement, the mould was compressed between two glass plates, covered with copier overhead film (Océ, MC 110, The Netherlands) to facilitate removal after hardening. The glass plates were manually compressed up to the time specified for final hardening, after which they are left in place for 24 h. The stainless-steel strips were subsequently removed and the cement blocks were gently punched out of the mould. This yielded cement blocks with a central gap with a surface area of 0.61 cm^2 ^and a volume of 6 μl, as detailed previously [9]. The mean mass of each cement block was 200 mg. The blocks were macroscopically examined, and those with visibly entrapped gas bubbles very close to the surface or deviating weights were discarded.

### Gentamicin release in the interfacial gap model, maintained at 37°C

The first phase of the experiments, only the gaps in five sample blocks for each bone cement with 6 μL of PBS using a standard pipette. Capillary forces spontaneously contained the fluid inside the gap. After 5, 15, 30, 60, 120, and 240 min in a humid environment (relative humidity was approximately 90%), a sample block was taken out and the gap aspirated using a strip of filtration paper (Schleicher & Schuell, No. 602 h, Germany). Subsequently the filtration paper was put in 5 mL of phosphate buffered saline (PBS) and, after 24 h, an aliquot was taken out and stored at 4°C for later measurement of its gentamicin concentration.

**Figure 1:**
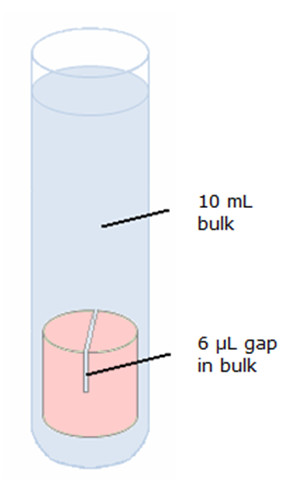
**Schematic presentation of gentamicin release into the filled gap with the possibility of further diffusion into the bulk fluid**. The outer surface of the sample block was effectively covered by nail varnish (indicated by the pink color) to prevent gentamicin elution from other surfaces than the gap.

In the second phase, the outer surface of fresh sample blocks for each bone cement was coated with three layers of a commercially available red nail polish. Each layer was left to dry for 1 h before application of the next layer. Three layers of the nail polish fully inhibited gentamicin elution for at least 1 week. By submersing the coated sample blocks in a larger fluid volume, thereby allowing gentamicin to diffuse from the gap into this larger volume, we simulate the clearance of gentamicin from the gap between bone and bone cement toward the serum, as occurring in a clinical situation [9]. The gaps of these blocks were again filled with 6 μL of PBS, after which the entire block was submersed in a bulk volume of 10 mL of PBS (see Figure 1). At 1, 6, 24, 48, 72, and 168 h, an aliquot (500 μL) of the bulk fluid was taken. Aliquots were stored at 4°C prior to measuring their gentamicin concentration.

Gentamicin concentrations were measured using a procedure introduced by Sampath and Robinson [13] and modified by Zhang et al. [14]. Briefly, an *o-*phtaldialdehyde reagent was made and stored for 24 h in a dark environment. The gentamicin sample, *o-*phtaldialdehyde, and isopropanol (to avoid precipitation of the products formed) were mixed in equal proportions and stored for 30 min at room temperature. The *o-*phtaldialdehyde reacted with the gentamicin amino groups and chromophoric products were obtained, whose absorbances were measured at 332 nm using a spectrophotometer (Spectronic^® ^20 Genesys™; Spectronic Instruments, Rochester, NY, USA). The gentamicin percentages released, relative to the total amount incorporated, were calculated for all cements used.

The gentamicin release experiments were performed in triplicate and a statistical analysis was performed in order to compare the release rates of each sample type. To this end, the Student's t-test for independent samples was used. A 95% (p < 0.05, two-tailed) confidence interval was applied for statistical significance.

### Antibacterial efficacy of gentamicin eluent in the interfacial gap model

For bacterial growth, two bacterial strains were used: a gentamicin-sensitive CNS 7319 (Coagulase Negative Staphylococci 7319; MIC gentamicin = 0.047 μg/mL) and a gentamicin-resistant CNS 5147 (MIC gentamicin >256 μg/mL). The MIC of gentamicin was determined by using Etest strips (AB bioMérieux, Solna, Sweden). Both strains were clinical isolates retrieved from infected joint prostheses and cultured from cryo preservative beads (Protect Technical Service Consultants Ltd., Lancashire, UK) onto blood agar plates at 37°C in ambient air for 24 h. One colony from this plate was used to create a pre-culture in 10 mL Tryptone Soy Broth (TSB, Oxoid, UK) under the same incubating conditions, yielding a mean growth density after 24 h for both bacteria of 4.7 × 10^8 ^CFU/mL, as determined by counting the number of colony forming units (CFU) after growth of serial dilutions on TSB agar plates. This pre-culture was subsequently diluted in TSB at 1:10, to provide new nutrients, prior to filling the gaps in the bone cement with 6 μL of this dilution. These inoculated bone cement blocks were incubated for 24 h in a water vapour saturated environment at 37°C before microbiological evaluation.

After bacterial growth in the gaps, the bone cement blocks were broken to expose the gap surface and both sides were scraped with a stainless steel surgical blade to harvest the bacteria adhering to the biomaterial surface. Complete removal of adhering bacteria was occasionally verified using a confocal laser scanning microscope (Leica TCS-SP2; Leica Microsystems Heidelberg GmbH, Heidelberg, Germany). Broken cement blocks were stained with live/dead bacterial viability stain after scraping and examined for adhered bacteria. As there were no green (living) or red (dead) bacteria seen to be left on the cement surface, it can be safely assumed that scraping removed all adhered bacteria. The blade was wiped with a cotton swab, soaked in 9 g/L sodium chloride, which was then put in 4.5 mL of 9 g/L sodium chloride, vortexed, and sonicated for 60 s in a 35 kHz ultrasonic bath (Transsonic TP 690-A, Elma^®^, Germany). Serial dilutions were made and poured on TSB agar plates for overnight incubation at 37°C and enumeration the next day.

All results were expressed in ^10^log CFU and experiments were carried out in triplicate with separately cultured strains, unless bacterial growth was fully absent on the gentamicin-loaded variant in the first two experiments in which case the experiment was only performed twice. To determine effects of the gentamicin release on bacterial survival and growth, ^10^log CFU values for gaps in gentamicin-loaded bone cements were compared, employing a two-tailed Student's t-test for unpaired samples.

## Results

### Cement powder characteristics

**Figure 2:**
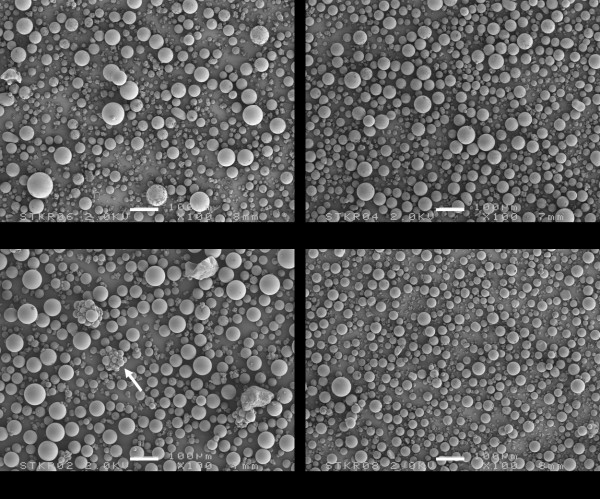
**SEM micrographs of the Refobacin Palacos R, Refobacin Bone Cement R, Palacos R + G, and SmartSet GHV powder**. The arrow indicates agglomerates of the smaller radiopacifier particles. Bar denotes 100 μm.

**Figure 3:**
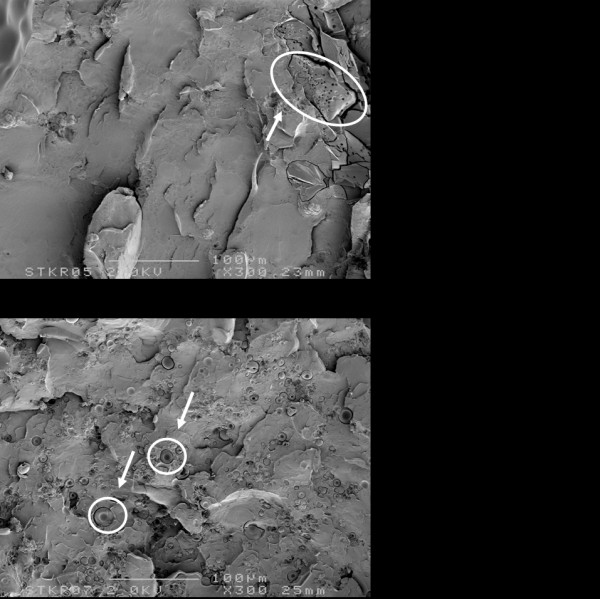
**SEM micrographs of the fractured surface of cured specimens of Palacos R + G and SmartSet GHV bone cement**. Note the difference in gentamicin particle shape, indicated by arrows.

**Figure 4:**
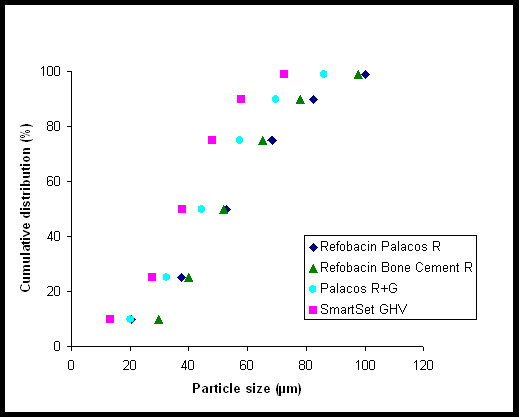
**Powder particle size distributions of the Refobacin Palacos R, Refobacin Bone Cement R, Palacos R + G, and SmartSet GHV powder**.

Each cement powder consists of a mixture of different components (Figure 2). The larger and prevailing structures observed in all cement powders are spherical granules corresponding to pre-polymerized PMMA and the size of these beads varies between 10 μm and 100 μm. The remainder of the structures observed are much smaller and correspond with radio-pacifiers (about 15 w/w% zirconium dioxide) and 2-4 wt% gentamicin particles. Zirconium dioxide particles are more or less polyhedral with a size range between 1 μm to 5 μm, are added to facilitate X-ray contrast. A problem associated with the use of 1-5 μm diameter radio-pacifier particles is that incomplete dispersion of the particles may result in the formation of particle agglomerates of 50 μm - 200 μm in diameter (Figure 2). The gentamicin particles (with diameter of 5 μm - 40 μm) in cured SmartSet GHV clearly appear as spherical particles (Figure 3), while the Palacos variants include much larger antibiotic particles with a more crystalline structure (Figure 3). The particle distributions of the cement powders are shown in Figure 4. Two features are noted: (a) the proportion of small-sized PMMA beads (mean diameter, d, between 5 μm and 40 μm) in the powder, and (b) the proportion of large-sized PMMA beads (d ≥ 75 μm) in the powder (Table 1). All Palacos variants contain large-sized PMMA beads (portion between 10-15 wt%), while SmartSet GHV only contained small-sized particles.

**Table 1:** Portion of small-sized (diameter 5 μm - 40 μm) and large-sized (diameter >75 μm) PMMA beads

Bone cements	small-sized beads (wt%)	large-sized beads (wt%)
Refobacin Palacos R	30 ± 0	15 ± 0

Refobacin Bone Cement R	25 ± 1	10 ± 1

Palacos R + G	40 ± 0	10 ± 0

SmartSet GHV	55 ± 0	0 ± 0

Proportions were determined using the numbers presented in Figure 4, with ± indicating the approximate accuracy of the determination.

### Wettability

Water contact angles on the different bone cements ranged between 65 and 70 degrees, with no significant differences between the four cements.

### Gentamicin release characteristics

**Figure 5:**
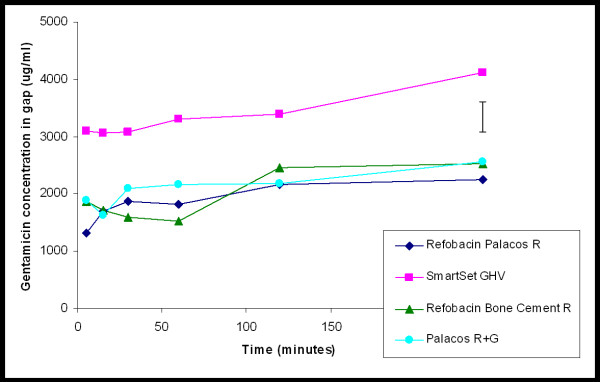
**Gentamicin concentration as a function of time of exposure to 6 μL of phosphate-buffered saline in a gap**. The values are expressed as mean of three separate experiments, error bar denotes the average standard deviation.

In Figure 5, the patterns of release of gentamicin from the cements into the gap, when only the gap is filled with fluid, are presented. The initial release rate of SmartSet GHV cement is much higher than of the other cements. SmartSet GHV cement rapidly obtained a high gentamicin release during the first 30 min after the start of an experiment and demonstrates the highest cumulative release in the first 4 h. The total release into the gap after 4 h, expressed relative to the amount of gentamicin incorporated, is 0.8 ± 0.2%, 0.9 ± 0.3%, 1.0 ± 0.7%, 0.7 ± 0.2% for the Refobacin Palacos R, Refobacin Bone Cement R, Palacos R + G, and SmartSet GHV, respectively. The minor differences in relative release may indicate that the higher initial release of SmartSet GHV is the result of the higher gentamicin content in this cement.

**Figure 6:**
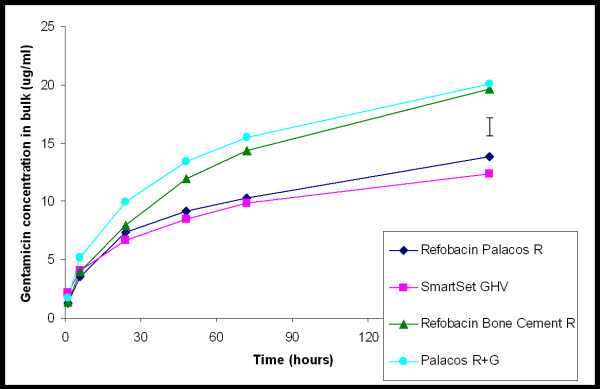
**Gentamicin concentration as a function of time of exposure of a gap to 10 mL of phosphate buffered saline**. The values are expressed as mean of three separate experiments, error bar denotes the average standard deviation.

The patterns of cumulative gentamicin release from the cement into the bulk fluid, are shown in Figure 6. Besides differences in the cumulative amounts of gentamicin released, differences are also seen in the kinetics of gentamicin release. The release of gentamicin from SmartSet GHV and Refobacin Palacos R increases somewhat less after prolonged release than from Palacos R + G and Refobacin Bone Cement R. Gentamicin release from Refobacin Bone Cement R and Palacos R + G is significantly more rapid, statistical significant increase (p < 0.05), than the release of gentamicin from SmartSet GHV and Refobacin Palacos R. After 1 week 8.6 ± 0.6%, 12.2 ± 0.8%, 12.5 ± 3.6%, 3.6 ± 0.4% of the total gentamicin content of a sample block was released for the Refobacin Palacos R, Refobacin Bone Cement R, Palacos R + G, and SmartSet GHV, respectively.

### Antibacterial efficacy of gentamicin eluent

The mean numbers of the colony-forming units harvested from the bone cement surfaces constituting the gap when placed in a water saturated environment, i.e. when only the gap is filled with fluid, are summarised in Table 2. Sizeable numbers of bacteria were found on all bone cements for the gentamicin-resistant strain (mean ^10^log CFU ranged from 4.3 for Refobacin Palacos R to 4.6 for SmartSet GHV) and the small differences were not statistically significant (p > 0.05). The gentamicin-sensitive strain was unable to survive in gaps made in all four gentamicin-loaded cements (Table 2).

**Table 2:** Clinically isolated staphylococcal strains used in this study with their gentamicin susceptibility and inoculum size (^10^log CFU)

Bacterial strain	Gentamicin susceptibility	Inoculum size	Refobacin Palacos R	Palacos R + G	Refobacin Bone Cement R	SmartSet GHV
CNS 7319	Sensitive	8.1 ± 0.7	-	-	-	-

CNS 5174	Resistant	9.3 ± 0.7	4.3 ± 0.4	4.4 ± 0.8	4.4 ± 0.9	4.6 ± 0.8

Also the mean number of colony forming units (^10^log CFU) harvested from gaps prepared in the four commercially available gentamicin-loaded bone cements are shown. Note that only the gaps were filled with fluid and not the volume above the samples. Results are means from three separate experiments (n = 3) ± SD, unless no growth.

- = no growth

## Discussion

The continuing emergence of new commercially-available brands of ALBCs makes it important to establish which one will provide the most favourable antibiotic release, and consequently yields the best antibacterial efficacy. An *in vitro *antibiotic release and antibacterial efficacy study was therefore carried out to compare SmartSet GHV, the original Palacos bone cement (previously marketed as Refobacin Palacos R), and its two follow-up products, Refobacin Bone Cement R and Palacos R + G.

In an ALBC, antibiotic release typically occurs in a biphasic manner, with most of the antibiotic being released in the first hours (burst release), and then continuing to release at low levels (sustained release). Our gap measurements only represent the burst release, because longer time intervals were impracticable due to evaporation of the very small volume (6 μL of PBS) inside the gap, and despite positioning of the experiment in a humid environment. SmartSet GHV showed a statistically significantly higher gentamicin release in the gap after 4 h when compared with the three Palacos variants. All three Palacos variants appeared to have similar burst releases. As the burst release is due to the dissolution of antibiotic particles at the surface, the amount of antibiotics released from bone cement is proportional to its initial concentration in the powder [15]. This is further corroborated by the small differences in relative amounts released from the three Palacos variants.

The bulk fluid volume above the gaps obtains its antibiotic concentration from diffusion out of the narrow gap. The gap, however, contains only very small amounts of antibiotic (about 20 μg in total, as can be derived from Figure 5), while total gentamicin amounts in the bulk fluid are tenfold higher (compare Figure 6). This implies that our bulk gentamicin measurements solely represent the sustained release of antibiotic out of the different cements, but cannot be related to a clinically relevant volume as the gap volume itself. There was no significant difference in gentamicin release measured in the bulk between Refobacin Bone Cement R and Palacos R + G, but Refobacin Palacos R released significantly less gentamicin. This trend is interesting because the three cements have almost identical compositions and both Refobacin Bone Cement R and Palacos R + G were introduced into the market as replacements for Refobacin Palacos R. Also, there was less gentamicin release from SmartSet GHV when compared to Refobacin Bone Cement R and Palacos R + G. This finding confirms previous results in which it was found that, over a period of 72 h, there was significantly more gentamicin release from Palacos R + G than from SmartSet GHV [6]. Ideally, gentamicin-loaded bone cements should present a high total release with sustained high concentrations of the antibiotic, especially because aminoglycosides, such as gentamicin, have a concentration dependent antibacterial activity [16]. Sustained high gentamicin release is, therefore, clinically desirable as therapeutic effectiveness of gentamicin will not continue once release rates fall below certain levels, possibly associated with the risk of inducing antibiotic resistance.

Sustained release requires the penetration of dissolution fluids into the interconnecting pores and cracks, which is dictated by the wettability of the polymer matrices and by the number and sizes of the pores in the polymer matrix. The wettability of a polymer matrix can be determined by measuring the water contact angles. If the water contact angle on a polymer surface decreases, it implies that the wetting of the surface is better and that solvent might penetrate more easily in pores and holes in the matrix to dissolve the antibiotic particles. Water contact angles of the four bone cements were between 65 and 70 degrees, i.e. all cements were equally hydrophobic, indicating that penetration will be equally slow for all cements. The differences in release kinetics observed can therefore only be explained by differences in the number and size of pores in the cements [17].

Sustained release of antibiotics from ALBCs is largely influenced by porosity of the cement [17], and an increased polymer-to-monomer ratio leads to increased porosity and release of antibiotic from the cement [18]. The polymer-to-monomer ratios of Refobacin Palacos R, Refobacin Bone Cement R, Palacos R + G and SmartSet GHV are 1.82, 1.83, 1.83 and 1.75, respectively. Although these differences are small, the higher amount of monomer in SmartSet GHV may cause a more closed matrix, explaining the lower sustained release. Moreover, a more closed matrix of Smartset GHV may be attributed to the large proportion of small-sized PMMA beads in its powder as compared to the case for the three Palacos variants.

In addition, large-sized PMMA beads will maintain their spherical form in the cured cement and will be responsible for a less dense matrix than can obtained with small-sized PMMA beads. Therefore, the PMMA size distribution will affect the gentamicin release. SmartSet GHV only contained small-sized particles, while all Palacos variants also contain large-sized PMMA beads (between 10% and 15%). Although the above described differences may appear minor, they greatly influence the density of the bone cement matrix and consequently the antibiotic release from it.

Both the dose and the duration of the gentamicin release are, in large part, strongly influenced by the size and shape of the antibiotic particles [19]. The gentamicin in Palacos R + G is coarser than in Refobacin Palacos R which, according to the manufacturer, stimulates more gentamicin release from the former cement [20]. Optimum release is achieved if the antibiotic is in the form of a crystalline formulation rather than a fine powder [18]. The dissolution of large crystalline structures from the surface of PMMA may be more rapid than that of the fine powder which may be more closely associated with the polymer. However, use of large antibiotic particles may compromise its mechanical strength by increasing porosity. SmartSet GHV cement is therefore loaded with small sized antibiotic particles [21]. These particles diffuse in a controlled manner from the entire cement matrix with minimal effect on the mechanical stability of the cement.

The extent to which an antibiotic prevents the formation of a biofilm is an index of its efficacy. Therefore, differences in biofilm formation have also been investigated for the four gentamicin-loaded bone cements in an *in vitro *simulation model of the prosthesis-related interfacial gap [10]. Bacterial killing in the gaps occurred only with the gentamicin-sensitive strain, although the concentrations of gentamicin found inside the isolated gaps went up to 4000 μg/ml within 4 h. These concentrations may be expected to effectively decontaminate the prosthesis-related interfacial gap directly after implantation, provided that the bacteria are sensitive for gentamicin, but appeared insufficient to kill the gentamicin-resistant strain. Indeed, the gentamicin concentrations found inside the isolated gaps were only about 10 times higher than the MIC of the resistant strain determined by using Etest strips, which ignores the additional resistance created by the biofilm mode of growth occurring in the gap model. Consequently, all four gentamicin-loaded bone cements showed bacterial growth of the resistant strain inside the isolated gap and there was no significant difference in bacterial survival despite the significant higher burst release seen with SmartSet GHV. Differences in antimicrobial efficacy might become apparent if a strain with an MIC close to the observed gentamicin concentrations was selected. In our case, these concentrations were probably well above or well below the required levels to kill the particular strain, for the sensitive and resistant strain, respectively.

## Conclusions

Although marketed as the original Refobacin Palacos R, orthopaedic surgeons should be aware that the successor cements (Refobacin Bone Cement R and Palacos R + G) do not appear to have the same release characteristics as the original cement. All Palacos variants showed equal burst releases, but each of the successor cements showed significantly greater sustained releases. SmartSet GHV showed a significantly higher burst release, while its sustained release was comparable with original Palacos. Overall, high gentamicin concentrations were reached inside our prosthesis-related interfacial gaps. These concentrations may be expected to effectively decontaminate the prosthesis-related interfacial gap directly after implantation, provided that these bacteria are sensitive for gentamicin, as a gentamicin-sensitive bacterium did not survive in the interfacial gap over a time period of 24 h, irrespective of the antibiotic-loaded bone cement involved. A gentamicin-resistant strain did survive in the interfacial gaps, regardless of the type of bone cement used. Survival rates were independent of the level of burst release by the bone cement.

## Competing interests

Since 2005, the authors have received reimbursements from DePuy International Ltd. for this manuscript but they do not hold any stocks or shares or patents relating to the content of the manuscript. The authors do not have any non-financial competing interests.

## Authors' contributions

DN conceived of the study, and participated in its design and coordination and drafted the manuscript. OK carried out the gentamicin release and bacterial growth studies, and performed the statistical analysis. JT, HvdM and HB participated in the design of the study and helped to draft the manuscript. All authors read and approved the final manuscript.

## Pre-publication history

The pre-publication history for this paper can be accessed here:

http://www.biomedcentral.com/1471-2474/11/258/prepub

## References

[B1] Neut D Van de Belt H Van Horn JR Van der Mei HC Busscher HJ The effect of mixing on gentamicin release from polymethylmethacrylate bone cements Acta Orthop Scand 2003 74 670 676 10.1080/00016470310018180 14763697

[B2] Liu CZ Green SM Watkins ND Baker D McCaskie AW Dynamic creep and mechanical characteristics of SmartSet GHV bone cement J Mater Sci Mater Med 2005 16 153 160 10.1007/s10856-005-5893-y 15744604

[B3] Dall GF Simpson PM Mackenzie SP Breusch SJ Inter- and intra-batch variability in the handling characteristics and viscosity of commonly used antibiotic-loaded bone cements Acta Orthop 2007 78 412 420 10.1080/17453670710014004 17611857

[B4] Dall GF Simpson PM Breusch SJ In vitro comparison of Refobacin-Palacos R with Refobacin Bone Cement and Palacos R + G Acta Orthop 2007 78 404 411 10.1080/17453670710013997 17611856

[B5] Bridgens J Davies S Tilley L Norman P Stockley I Orthopaedic bone cement: do we know what we are using? J Bone Joint Surg [Br] 2008 90 643 647 18450633 10.1302/0301-620X.90B5.19803

[B6] Simpson PM Dall GF Breusch SJ Heisel C [*In vitro *elution and mechanical properties of antibiotic-loaded SmartSet HV and Palacos R acrylic bone cements] Orthopade 2005 34 1255 1262 10.1007/s00132-005-0861-2 16136337

[B7] McLaren RL McLaren AC Vernon BL Generic tobramycin elutes from bone cement faster than proprietary tobramycin Clin Orthop Relat Res 2008 466 1372 1376 10.1007/s11999-008-0199-2 18340503 PMC2384044

[B8] Dunne N Hill J McAfee P Todd K Kirkpatrick R Tunney M Patrick S *In vitro *study of the efficacy of acrylic bone cement loaded with supplementary amounts of gentamicin: effect on mechanical properties, antibiotic release, and biofilm formation Acta Orthop 2007 78 774 785 10.1080/17453670710014545 18236183

[B9] Hendriks JG Neut D Van Horn JR Van der Mei HC Busscher HJ The release of gentamicin from acrylic bone cements in a simulated prosthesis-related interfacial gap J Biomed Mater Res B Appl Biomater 2003 64 1 5 10.1002/jbm.b.10391 12474240

[B10] Hendriks JGE Neut D Van Horn JR Van der Mei HC Busscher HJ Bacterial survival in the interfacial gap in gentamicin-loaded acrylic bone cements J Bone Joint Surg [Br] 2005 87 272 276 15736756 10.1302/0301-620x.87b2.14781

[B11] Wang JS Franzen H Lidgren L Interface gap after implantation of a cemented femoral stem in pigs Acta Orthop Scand 1999 70 234 239 10.3109/17453679908997799 10429597

[B12] Gristina AG Costerton JW Bacterial adherence to biomaterials and tissue. The significance of its role in clinical sepsis J Bone Joint Surg [Am] 1985 67 264 273 3881449

[B13] Sampath SS Robinson DH Comparison of new and existing spectrophotometric methods for the analysis of tobramycin and other aminoglycosides J Pharm Sci 1990 79 428 431 10.1002/jps.2600790514 2352163

[B14] Zhang X Wyss UP Pichora D Goosen MF Biodegradable controlled antibiotic release devices for osteomyelitis: optimization of release properties J Pharm Pharmacol 1994 46 718 724 7837040 10.1111/j.2042-7158.1994.tb03890.x

[B15] He Y Trotignon JP Loty B Tcharkhtchi A Verdu J Effect of antibiotics on the properties of poly(methylmethacrylate)-based bone cement J Biomed Mater Res 2002 63 800 806 10.1002/jbm.10405 12418027

[B16] Lacy MK Nicolau DP Nightingale CH Quintiliani R The pharmacodynamics of aminoglycosides Clin Infect Dis 1998 27 23 27 10.1086/514620 9675444

[B17] Van de Belt H Neut D Uges DR Schenk W van Horn JR van der Mei HC Busscher HJ Surface roughness, porosity and wettability of gentamicin-loaded bone cements and their antibiotic release Biomaterials 2000 21 1981 1987 10.1016/S0142-9612(00)00082-X 10941919

[B18] Downes S Methods for improving drug release from poly(methyl)methacrylate bone cement Clin Mater 1991 7 227 231 10.1016/0267-6605(91)90063-L 10149136

[B19] Park JB Lakes RS Biomaterials: An Introduction 1992 2 Plenum Publishing Corporation, New York 29 62

[B20] Palacos^® ^brochure http://www.palacos.com.cn/fileadmin/user_upload/documents/EN/PALACOS_RevUK060220.pdf (date last accessed 28 May 2010)]

[B21] SmartSet GHV brochure http://www.depuyorthopaedics.com/HealthCare/Pages/HealthCareProductResults.aspx?CategoryName=O.R.%20Cement%20and%20Accessories&GroupName=Bone%20Cement%20and%20Accessories&Product=SmartSet%c2%ae%20GHV%20Bone%20Cement&CN=O.R.%20Cement%20and%20Accessories&GN=Bone%20Cement%20and%20Accessories&PN=SmartSet%c2%ae%20GHV%20Bone%20Cement (date last accessed 28 May 2010)]

